# Autism Linked to Increased Oncogene Mutations but Decreased Cancer Rate

**DOI:** 10.1371/journal.pone.0149041

**Published:** 2016-03-02

**Authors:** Benjamin W. Darbro, Rohini Singh, M. Bridget Zimmerman, Vinit B. Mahajan, Alexander G. Bassuk

**Affiliations:** 1 Department of Pediatrics, Division of Medical Genetics, University of Iowa, Iowa City, Iowa, United States of America; 2 Interdisciplinary Program in Genetics, University of Iowa, Iowa City, Iowa, United States of America; 3 Roy J. and Lucille A. Carver College of Medicine, University of Iowa, Iowa City, Iowa, United States of America; 4 The Holden Comprehensive Cancer Center, University of Iowa, Iowa City, Iowa, United States of America; 5 Department of Pediatrics, Division of Pediatric Hematology/Oncology/BMT, University of Iowa, Iowa City, Iowa, United States of America; 6 Department of Biostatistics, University of Iowa College of Public Health, Iowa City, Iowa, United States of America; 7 Department of Ophthalmology and Visual Sciences, University of Iowa, Iowa City, Iowa, United States of America; 8 Department of Biology, University of Iowa, Iowa City, Iowa, United States of America; 9 Department of Pediatrics, Division of Neurology, University of Iowa, Iowa City, Iowa, United States of America; 10 Interdisciplinary Graduate Program in Molecular and Cellular Biology, University of Iowa, Iowa City, Iowa, United States of America; 11 Interdisciplinary Graduate Program in Neuroscience, University of Iowa, Iowa City, Iowa, United States of America; 12 University of Iowa eHealth and eNovation Center, University of Iowa, Iowa City, Iowa, United States of America; King Faisal Specialist Hospital and Research center, SAUDI ARABIA

## Abstract

Autism spectrum disorder (ASD) is one phenotypic aspect of many monogenic, hereditary cancer syndromes. Pleiotropic effects of cancer genes on the autism phenotype could lead to repurposing of oncology medications to treat this increasingly prevalent neurodevelopmental condition for which there is currently no treatment. To explore this hypothesis we sought to discover whether autistic patients more often have rare coding, single-nucleotide variants within tumor suppressor and oncogenes and whether autistic patients are more often diagnosed with neoplasms. Exome-sequencing data from the ARRA Autism Sequencing Collaboration was compared to that of a control cohort from the Exome Variant Server database revealing that rare, coding variants within oncogenes were enriched for in the ARRA ASD cohort (p<1.0x10^-8^). In contrast, variants were not significantly enriched in tumor suppressor genes. Phenotypically, children and adults with ASD exhibited a protective effect against cancer, with a frequency of 1.3% vs. 3.9% (p<0.001), but the protective effect decreased with age. The odds ratio of neoplasm for those with ASD relative to controls was 0.06 (95% CI: 0.02, 0.19; p<0.0001) in the 0 to 14 age group; 0.35 (95% CI: 0.14, 0.87; p = 0.024) in the 15 to 29 age group; 0.41 (95% CI: 0.15, 1.17; p = 0.095) in the 30 to 54 age group; and 0.49 (95% CI: 0.14, 1.74; p = 0.267) in those 55 and older. Both males and females demonstrated the protective effect. These findings suggest that defects in cellular proliferation, and potentially senescence, might influence both autism and neoplasm, and already approved drugs targeting oncogenic pathways might also have therapeutic value for treating autism.

## Introduction

High-throughput DNA sequencing is dramatically increasing the availability of genome-scale sequencing data from patients. As a result, candidate disease genes are being implicated in more than one disorder, including neurodevelopmental disorders. The same genes are repeatedly implicated in intellectual disability (ID) [1], autism spectrum disorder (ASD) [2–4], epilepsy [5], and schizophrenia, an observation not altogether unexpected since these conditions all affect the brain. More surprisingly, however, many genes important in neurodevelopment are also implicated as potential drivers of neoplastic disease.

This observation is puzzling, but not entirely novel. Indeed, many of the genes implicated in causing hereditary tumor syndromes overlap with those involved in syndromic causes of neurodevelopmental disorders. *PTEN*, for example, is a well-known tumor suppressor gene somatically mutated in various cancers [6]. Germline loss-of-function *PTEN* mutations cause Cowden, Bannayan–Riley–Ruvalcaba, and other syndromes [7–11], and increase the rate of benign and malignant tumors. Interestingly, some *PTEN* mutations also manifest as ASD and macrocephaly [12]. Similarly, mutations in *TSC1* and *TSC2* genes cause tuberous sclerosis complex which is characterized by cortical tubers, and neurocognitive phenotypes including epilepsy, ASD, and ID [13–15]. Initially, the neurocognitive abnormalities were attributed to cortical tubers, but recent research found patients also show extensive, microscopic brain changes and disrupted brain architecture outside of the cortex; and some patients have no tubers but are still neurocognitively abnormal [14]. Similarly, *NF1* mutations, other “RAS-opathies” and *APC* mutations (especially deletions) cause familial tumor syndromes, but can also confer ID and ASD phenotypes [16,17].

Recently, the list of such examples has grown to include *ATRX*, *BCOR*, *BRAF*, *CREBBP*, *CTNNB1*, *KDM5C*, *MED12*, *MET*, *PHF6*, *PTPN11*, *EP300*, *SMARCB1*, and *MLL2 [3,18–36].* Copy number variants (CNVs) also contribute to this list as several CNVs believed to underlie neurodevelopmental disorders also contain cancer predisposition genes [37]. These observations are the foundation for this study, which sought to define the relationship between neurodevelopmental disorders, specifically ASD, and neoplastic disease at genotypic and clinical phenotypic levels.

## Methods

### Cancer gene selection

Oncogenes and tumor suppressors were selected based on 1) the Sanger Cancer Gene Census [38]; 2) the Cancer Genome Landscapes review [39] by Vogelstein et al., 2013; and 3) the CancerGenes website, manually curated by Walker et al. [40]. Genes appearing in at least two sources were selected (S1 Table). Genes for other conditions tested, including ASD, intellectual disability, epilepsy, skeletal dysplasia, dilated cardiomyopathy, retinitis pigmentosa, and non-syndromic hearing loss were obtained by comparing several clinically available multi-gene sequencing panels (S1 Table).

### Variant enrichment analysis

The ARRA Autism Sequencing Collaboration (dbGaP Study Accession: phs000298.v1.p1) [41] provided exome sequencing data on the frequency of rare, nonsynonymous, coding, single-nucleotide variants in ASD patients. Only single-nucleotide variation (SNV) data generated by the Broad Institute were used. VCFtools was used to filter the Broad-generated VCF file (c1_NIMH_Autism_HGSC_Broad_broad.vcf), to select only individuals with an autism diagnosis (excluding non-affected parental samples, S2 Table) and only the genomic coordinates of selected genes [42]. Genomic coordinates (hg19) for gene coding sequence, plus 25 nucleotides on either side, were obtained from the UCSC Genome Browser using the Table Browser tool [43]. Our control dataset was from the Exome Variant Server (Exome Variant Server, NHLBI GO Exome Sequencing Project (ESP), Seattle, WA (URL: http://evs.gs.washington.edu/EVS/) [2/2014 accessed]. A composite VCF file was downloaded from the EVS website, which included variant results from 6503 individuals. Indels were removed from the EVS dataset, to compare it to the Broad autism dataset, which included only SNVs. Numerous quality control measures were used to account for the potential technical artifacts of comparing two cohorts sequenced at different times, places, and under different conditions. Depth filters were applied to both datasets. VCFtools filtered out variant sites in the autism cohort with a mean depth value of less than 10x in samples where a genotyping call was made. At genomic positions in the EVS dataset, depth of coverage was retrieved from the EVS website and used to filter out variant positions with a mean depth value of less than 10x. Variant sites within the autism cohort were removed if they fell within exome regions where the EVS dataset had less than 10x average coverage. Variants were filtered out if they occurred in annotated segmental duplications, within homopolymer regions (if the continuous homopolymer was longer than three repeated nucleotides), or had a minor allele frequency of >1% in either the 1000 Genomes Phase I data release [44], EVS (allele frequency for both populations), or >5% in any one population (as reported within dbSNPv142). dbSNPv142 variants annotated as “suspect” were excluded. Only variants predicted to change the coding sequence were retained; this included non-synonymous missense variations, nonsense, and splice-site variants. Variants were annotated with the GATK Variant Annotator and SnpEff [45–47]. Splice-site variants were those within six nucleotides of a splice site. VarSifter and several custom Python scripts were used for filtering [48]. Any variant within the ASD cohort at an allele frequency >5% (after filtering for only rare variation) was excluded because these are likely artifacts. This last filter also greatly reduces the likelihood that a single variant, or a few variants within a single gene, are disproportionately responsible for any enrichment signal observed.

Enrichment was determined by calculating the total number of SNVs within the gene set under investigation in each cohort. This number was divided by the number of “mean genotypable alleles” in our entire cohort. This corrects for variants at genomic locations where few autism cohort samples had enough sequencing depth to genotype them with confidence. This methodology accounts for what is likely an underestimate of variant alleles at some poorly covered sites. The same approach was used with the data from the EVS. For example, whereas the autism cohort contained 515 samples (or a maximum of 1,030 possible alleles per variant site), the mean of the actual number of alleles genotyped was lower (771 for oncogenes and 772 for tumor suppressor analysis). The same approach was used with the data from the EVS, where the maximum number of alleles per variant site was 13,006 but the mean of the actual number of alleles genotyped was also lower (12,827 for the oncogene analysis and 12,700 for the tumor-suppressor analysis). Significance and p-values were calculated by chi-squared analysis (2x2 contingency table).

### EMR query and epidemiologic analysis

This study was approved by the University of Iowa Institutional Review Board. Data concerning University of Iowa Hospitals and Clinics (UIHC) patients was obtained from our EMR (EPIC; Verona, WI), using the clinical informatics program Starmaker (Park Street Solutions; Naperville, IL). Records were from the outpatient, emergency room, and inpatient settings from 2009 to March 2015. Patient information was de-identified prior to analysis, and written consent from the participants was not obtained due to the retrospective nature of the study and anonymity of the patient records. Among UIHC patients, 1,837 had the diagnosis of autism, (“autistic disorder” in our EMR, ICD-9 code 299.0). Patients were cataloged for the presence and type of a neoplasm (ICD-9 codes 140 to 239.99). Randomly selected control patients had any diagnosis except ASD, and were generated via the same program. Each patient was assessed for a diagnosis of neoplasm. In each combined age and gender category, control patients outnumbered the ASD cohort by ~5:1. These autistic patients and additional randomly selected control patients were analyzed for the diagnoses of diabetes mellitus (ICD-9 code 250) and essential hypertension (ICD-9 code 401). Initial descriptive statistics were analyzed using SPSS version 22 (SPSS Inc.; Chicago, IL). Logistic regression was performed using SAS version 9.4 (SAS Institute; Cary, NC). The logistic model examined the diagnosis of neoplasm as the dependent variable and the diagnosis of autistic disorder as the independent variable. The model included age, gender, and their interaction with autistic disorder. Odds ratios and 95% confidence intervals were computed.

Additional analysis examined patients with the diagnoses of atopic dermatitis (ICD-9 code 691), esophageal reflux (ICD-9 code 530.81), allergic rhinitis (ICD-9 code 477), and short stature (ICD-9 0.054). A set of randomly selected patients, the same size as the ASD cohort, with any diagnosis was generated. Patients with the diagnosis of ASD were excluded in all of these cohorts. To the extent possible, patients with each disease were matched by age and gender to the ASD cohort. Each patient within each of these case cohorts was indexed for the diagnosis of neoplasm. The control group was another large randomly generated group of patients with any diagnosis (but ASD), matched exactly by age range and gender. Logistic regression was performed with neoplasm as the dependent variable and atopic dermatitis, esophageal reflux, allergic rhinitis, and short stature were independent variables. Logistic regression was also performed to compare the cohort of 1,837 patients with any diagnosis (except ASD) to the larger control group.

## Results

### Enrichment for oncogene variants in autistic cohort

To investigate the genetic relationship between autism and cancer, a previously sequenced autism cohort was examined for the frequency of variants in oncogenes and tumor suppressors. In both autism and control cohorts, well-established oncogenes and tumor suppressors (see Methods) were identified and scanned for rare (<1% MAF), coding (non-synonymous, nonsense, splice site), single-nucleotide variations (SNVs). In addition to population frequency and coding status, variants were filtered to ensure quality control (see Methods). Sequence data for the autism cohort was originally generated by the ARRA Autism Sequencing Collaboration (dbGaP Study Accession: phs000298.v1.p1); and sequencing data for our control cohort was generated by the NHLBI GO Exome Sequencing Project (ESP) and deposited in the Exome Variant Server database [41].

Enrichment analysis examined the level of variation among our list of tumor suppressor genes and oncogenes. To calculate an enrichment value, the total number of rare, coding variants detected was divided by the average number of genotypable alleles for that cohort (see Methods and Table 1). Significance and p-values were calculated by chi-squared analysis. The number of rare, coding variants found within tumor suppressor genes was slightly greater in the autism cohort compared to the EVS control cohort but did not reach statistical significance (p = 0.20). In comparison, rare coding variants of oncogenes were over-represented in the autism cohort, reaching statistical significance (p<1.0x10^-8^).

**Table 1:** Variant enrichment analysis.

Gene List	ASD Alleles	Average Genotypable Alleles ASD	EVS Alleles	Average Genotypable Alleles EVS	ASD Enrich	EVS Enrich	P Value
Autism	1351	776	17507	12370	1.74	1.42	*9*.*28E-6*
Intellectual Disability	1961	769	25885	12098	2.55	2.14	*7*.*01E-05*
Epilepsy	882	774	10992	12760	1.14	0.86	*4*.*23E-08*
Oncogenes*	1305	771	16531	12827	1.69	1.29	*6*.*59E-09*
Tumor Suppressors	1275	772	19740	12700	1.65	1.55	0.2
Skeletal Dysplasia	2757	785	42846	12765	3.51	3.36	0.29
Dilated Cardiomyopathy	1994	789	30088	12395	2.53	2.43	0.36
Retinitis Pigmentosa	1307	760	23086	12650	1.72	1.82	0.21
Non-Syndromic Hearing Loss	1896	781	32183	12400	2.43	2.6	0.13
All RefSeq Genes	215209	779	3281990	12701	276.26	258.40	0.07

Variant enrichment in oncogenes, tumor suppressors, all RefSeq genes, and disease genes implicated in several other conditions in individuals with autism spectrum disorder (ASD) compared to controls (EVS). With a Bonferroni multiple comparison correction a p value of 0.005 (or 5.0E-3) is required to achieve statistical significance for any one of these ten gene lists.

* When missense variants predicted to be tolerated by MetaSVM [49] were removed the p value decreased to 2.26E-15.

Several additional gene lists were also analyzed for control purposes. Genes implicated in the disorders of autism, intellectual disability, and epilepsy were included as positive-control gene lists. Given the overlap in the lists of genes implicated in these neurodevelopmental disorders, it was not surprising that all three lists showed statistically significant enrichment for variants in the ASD cohort compared to the EVS control cohort (Table 1). To determine whether the enrichment for variants in ASD was specific to oncogenes and neurodevelopmental genes, as a negative-control we also analyzed lists of genes implicated in other disorders, including skeletal dysplasia, dilated cardiomyopathy, retinitis pigmentosa, and non-syndromic hearing loss. These lists did not overlap significantly with the neurodevelopmental gene lists. Statistical analysis suggested variants in the genes on those lists were not over-represented in ASD (Table 1). Since we used both positive and negative control gene lists, it is highly unlikely that the enrichment values observed for oncogenes and tumor suppressors are the result of a population matching error or other technical artifact. All variant lists are available in the Supporting Information (S1 and S2 Files). Synonymous SNVs within each gene list were not significantly enriched in the ASD cohort.

### Fewer cancers in second autistic cohort

**Fig 1:**
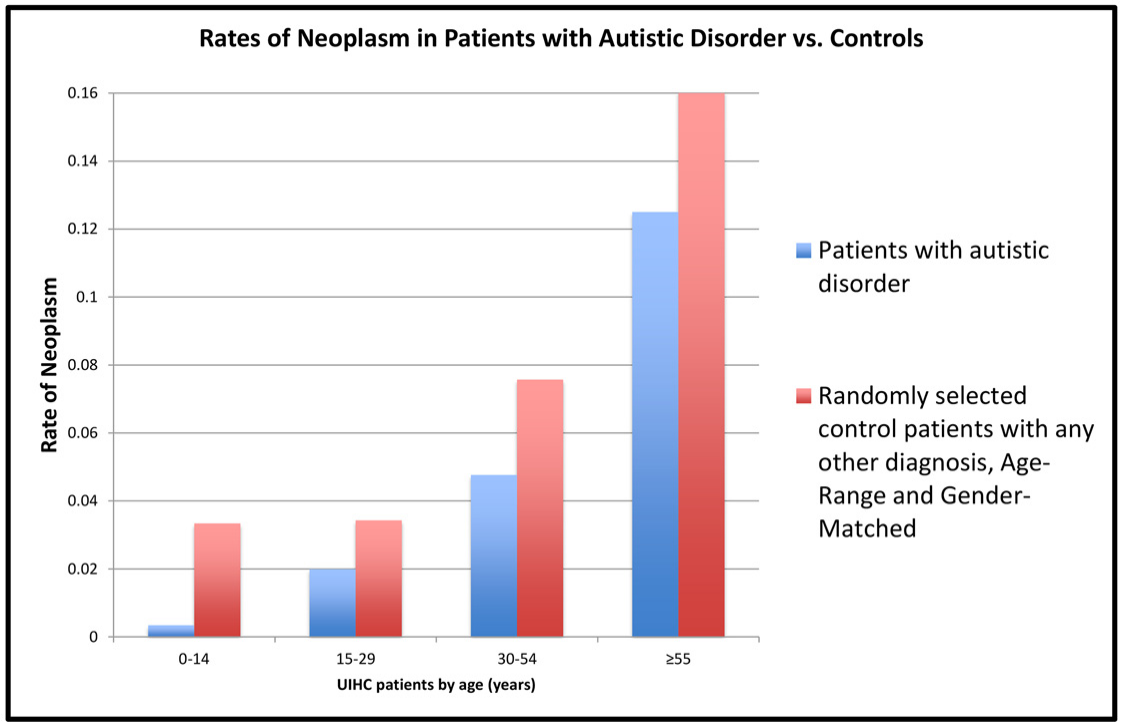
Cancer rates in patients diagnosed with autistic disorder and controls. Rates of neoplasm in patients at the University of Iowa Hospitals and Clinics with the diagnosis of autistic disorder (n = 1,837) vs. a control population, randomly selected patients with any diagnosis other than autistic disorder, matched to the patients with autistic disorder by age range and gender (n = 9,336). In every age range, patients with autistic disorder had lower rates of neoplasm compared to the control patients. In patients 0 to 14 years old, the control group had almost 10 times the rate of neoplasm compared to patients with autistic disorder. All of our regression models that include age and gender as well as interaction effects, show statistically significant effects of autistic disorder on the likelihood of developing a neoplasm.

Electronic medical record demographics of the autistic patients and controls are outlined in Table 2. Cancer was far less prevalent in autism patients as compared to controls (1.3% vs. 3.9%), with an odds ratio of neoplasm of 0.33 (95% CI: 0.22, 0.50; p<0.001). The differences were most profound among younger patients, with autistic disorder patients 0 to 14 years old having an almost 10 times lower rate of neoplasms (0.3%vs. 3.3%; Fig 1). In our study, 24 ASD patients were diagnosed with neoplasm, nine (~38%) with benign neoplasms and hemangiomas, and two with neurofibromatosis-related neoplasms (benign or malignant conditions were not specified). In the control patients, who did not have the diagnosis of ASD, the most commonly diagnosed neoplasms were consistent with the most common childhood cancers, including acute lymphoblastic leukemia and malignant brain tumors. Also, in the control patients, over 80% of the diagnosed neoplasms were malignant, pre-malignant, or of uncertain behavior (Table A in S3 File).

**Table 2:** Demographics of University of Iowa Hospitals and Clinics patients with autistic disorder.

	Patients with ASD	Controls
Total patients	1837	9336
Ages 0–14	1177	5301
Ages 15–29	502	2958
Ages 30–54	126	574
Ages ≥ 55	32	141
Males	1482	7562
Females	355	1774

**Fig 2:**
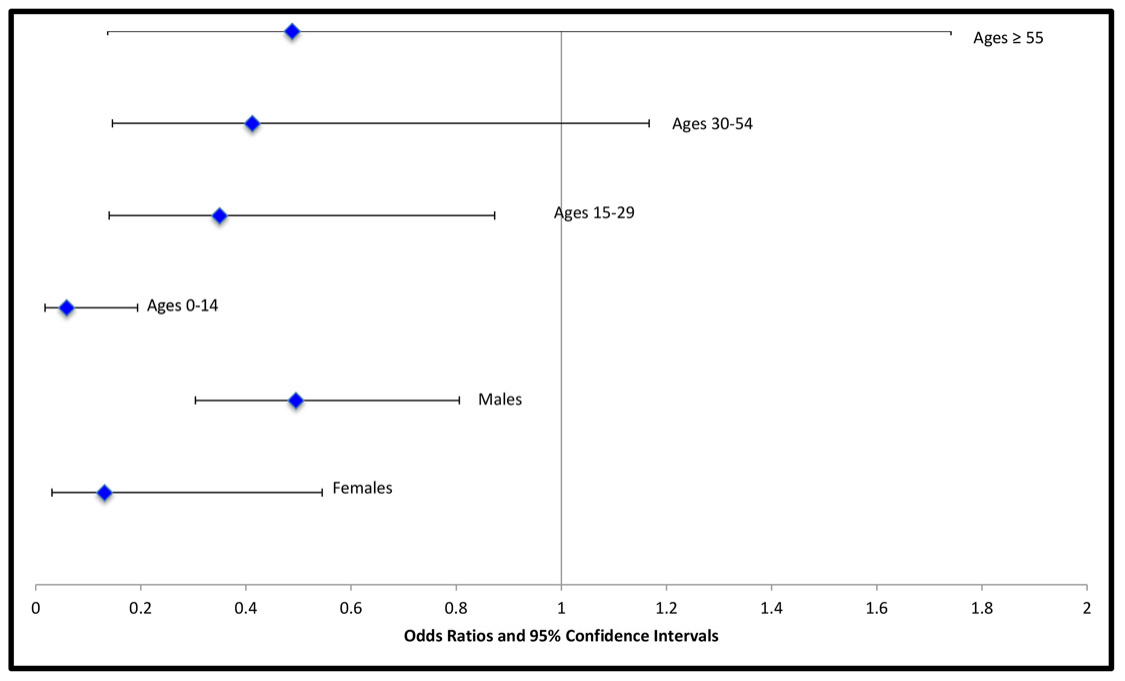
Effect of autistic disorder on cancer rate stratified by age and gender. The odds ratio of cancer for patients with autistic disorder relative to controls was modified by age and gender, with autism reducing the odds for neoplasm (compared to controls) more often in female patients, and more often in young patients.

Logistic regression analysis compared cancer rates between ASD patients and controls with any diagnosis except for ASD, and included age and gender (as well as the interaction of age and gender with autistic disorder), showing lower odds of neoplasms in ASD patients. The effect of autistic disorder on the odds of neoplasm decreased with increasing age (Fig 2; autism*age interaction p = 0.009). The odds ratio of neoplasm for those with autistic disorder relative to controls was 0.06 (95% CI: 0.02, 0.19; p<0.0001) in the 0 to14 age group, 0.35 (95% CI: 0.14, 0.87; p = 0.024) in the 15 to 29 age group; 0.41 (95% CI: 0.15, 1.17; p = 0.095) in the 30 to 54 age group; and 0.49 (95% CI: 0.14, 1.74; p = 0.267) in those 55 and older. Autism and gender appeared to interact (p = 0.080); compared to controls, odds ratios for neoplastic conditions with autistic disorder were 0.13 in females (95% CI: 0.03, 0.54; p = 0.005) and 0.50 in males (95% CI: 0.30, 0.81; p = 0.005; Table B in S3 File). The additive effect of autism with age and sex on the log-odds of neoplasm was quantified from this fitted logistic-regression model by computing odds ratios for each age-gender combination (Figure A and Table C in S3 File). The difference in the odds of neoplasm was greatest in females 14 years or younger (97% lower odds of neoplasm in those with autism); the smallest difference was in males 55 years and older, with an odds ratio of 0.95.

Patients over the age of 30 comprised less than 10% of our cohort, and did not have significantly different odds ratios for neoplastic conditions as compared to the control patients of the same age. Given a p value of 0.09, a larger cohort of autistic patients over the age of 30 might yield a statistically significant result.

In contrast to the negative association between autism and cancer, no significant associations were found between autism and other common systemic diseases, i.e., diabetes mellitus and essential hypertension (Figures B and C in S3 File).

**Fig 3:**
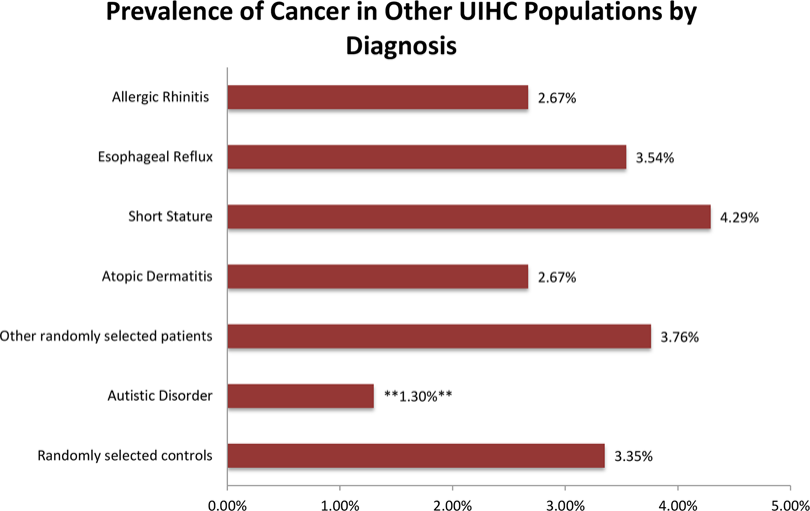
Cancer rates in other diagnoses. Prevalence of neoplasms in various diagnoses within the UIHC population compared to a control group of 9,336 patients with any diagnosis. When compared to controls, only autistic disorder showed a statistically significant difference in the odds for cancer.

Finally, logistic regression compared cancer rates between controls and patients with atopic dermatitis, esophageal reflux, allergic rhinitis, and short stature, but showed no statistically significant differences in odds for neoplasm (p values: atopic dermatitis: 0.162, esophageal reflux: 0.687, allergic rhinitis: 0.13, short stature: 0.054). The logistic regression analysis comparing the smaller group of randomly selected patients with any diagnosis to the control group also revealed no statistically significant differences in odds for neoplasm (p = 0.386; Fig 3 and Table D in S3 File).

## Discussion

We examined the link between autism and cancer by taking advantage of large existing databases of exome sequencing and EMR information. Our findings suggest autistic patients more often harbor rare, coding SNVs in oncogenes, yet tend to develop fewer neoplasms. These findings support recent reports that sets of genes are related to both cancer and neurodevelopment [50,51].

Some developmental theories of autism, such as the extreme male brain theory and the imprinted brain theory, predict that individuals with autism will have a higher risk of cancer [52–55]. These theories posit that increased cellular proliferation signals, due to either increased levels of sex steroids or imprinting defects, can dysregulate growth and trigger malignancy in mitotic cells, whereas in post-mitotic cells, such as neurons, they affect other growth properties (neurodevelopment). A small number of epidemiological studies also suggested either no change or a modest increase in the rate of particular cancer types in ASD (namely those of the central nervous and genitourinary system) [56–58]. Although the most recent of these studies employed an approach similar to ours (i.e., with diagnosis codes), it did not directly compare an autism cohort to a control population drawn from the same data source but instead used cancer incidence data from a national cancer registry.

Other studies might also seem to conflict with ours since they linked autism to changes in not only oncogenes but also tumor suppressors, including those involved in canonical cancer signaling pathways (e.g., the PI3K-AKT-PTEN-TSC-mTOR or PI3K-RAS-MAPK pathways), as well as chromatin-modifying proteins. This discrepancy likely reflects that the ARRA Autism Sequencing Collaboration cohort we used contains few syndromic cases (e.g., autistic patients with syndromic mutations in tumor suppressor genes, such as *NF1*, *PTEN*, *TSC1/2*). We focused on a general autistic patient population, finding that tumor suppressor genes were less enriched for rare, coding variation. Moreover, the results from our cohorts are consistent, since if ASD patients were also enriched for rare, coding variants of tumor suppressors (the majority of which would likely be damaging) the rate of cancer should be higher, not lower.

A more comprehensive evaluation of the oncogene and tumor suppressor variants found in this study is ongoing; nevertheless, the variations discovered in two oncogenes, *PIK3CA* and *MET*, implicate specific mechanisms that could link autism and neoplasm. For example, the ASD cohort had only two variations in the *PIK3CA* oncogene and both were in exon six (Y355F). Noticeably absent in the ASD and EVS cohort were well-known oncogenic variations in *PIK3CA* (i.e., E542K, E545K, and Q546K within exon 9 and H1047R within exon 20) [59]. If the *PIK3CA* and other oncogene variants found in the ASD cohort decrease signaling activity then these oncogenes in ASD patients may be “weaker” in fueling the growth-factor independent proliferation characteristic of neoplasms. Sequence variations outside of cancer “hot spots” (e.g., *PIK3CA* exon six) could reduce oncogenic function and thereby protect against malignancy in ASD patients [60]. We briefly explored this idea by repeating the enrichment analysis for oncogenes after removing missense variants predicted to be tolerated by the ensemble mutational impact score MetaSVM [49]. We found the enrichment for likely “damaging” (protein function altering) variants in oncogenes to be even more significant in the ASD cohort when compared to the EVS cohort (Table 1 footnote).

Another possible mechanism is illustrated by variations detected in the oncogene *MET*. Dysregulated signaling through MET is implicated in numerous malignancies of the gastrointestinal tract, genitourinary tract, breast, gynecologic organs, pulmonary system and skin [61]. DNA variations in *MET* are also implicated in autism and specifically in families with co-occurring autism and gastrointestinal conditions [62–64]. One mechanism that can be proposed for this pleiotropy is that *MET* variations associated with ASD appear to reduce the expression of MET (and presumably MET signaling) whereas variations associated with tumorigenicity increase MET tyrosine kinase signaling activity. We found 35 variations in *MET* within the ASD cohort and 598 within the EVS cohort. Some of the variations discovered are well recognized in the MET literature including the T1010I and R988C mutations [61]. In fact, 63% of the *MET* variations found in the ASD cohort were these two changes compared to only 25% in the EVS cohort. Interestingly, whereas these variations have been found in tumors and germlines of cancer patients and appear to increase MET signaling in vitro, they are not in the kinase domain itself, and there is considerable debate as to their transforming oncogenic capacity [61,65–69].

The data from *MET* illustrate how a continuum of activity may underlie the differential effects of oncogene mutations as they apply to either cancer or neurodevelopmental phenotypes. This phenomenon is further supported by data from the PI3K-AKT-mTOR pathway and specifically the tumor suppressor PTEN. Functional screening studies show *PTEN* variants common in patients with hereditary cancer syndromes reduce PTEN activity much more than variants commonly found in patients with ASD or DD [70].

Part of this continuum of activity may involve the process of cellular senescence. Here, it is notable that most oncogenes and tumor suppressor genes encode basic growth regulators expressed in the brain during early development [71]. Since the neuropathology of autism includes dysregulated neurogenesis and impaired neuronal migration, it is reasonable to believe both the ASD phenotype and the protective effect against cancer result from increased cellular senescence [72]. This idea is supported by the observations that autistic children have relatively shorter telomeres [73] and more neuronal senescence in the setting of reduced MECP2 (i.e., as in Rett syndrome) [74].

Neurodevelopment and oncogenesis are multi-step processes, and it is possible that signaling through the same cellular proliferation pathways can have different effects depending on embryological timing, as well as cell type, and mitotic status. Perhaps the most exciting implication here is that already interventions are underway to target cellular pathways shared by many of the mutated genes examined in this study. Thus, drugs known to treat cancer might also treat autism spectrum disorders in the future. In fact, several drugs, such as Rapamycin, have been developed to target and inhibit increased mTOR activity [75]. Rapamycin has been found not only to decrease tumor volume of subependymal astrocytomas, but also to prevent the development of autism in TSC-deficient mice [76]. A phase II trial in Boston is underway to study the benefits of a rapamycin derivative for patients with autism [77]. Another rapamycin derivative, everolimus, is benefiting patients with epilepsy through seizure reduction [78].

## Conclusions

Patients diagnosed with autism spectrum disorder have increased rare, coding variation in oncogenes yet decreased rates of cancer compared to controls.

This work was previously presented as an abstract at the 3rd Biennial Conference on Pediatric Neuro-Oncology Basic and Translational Research [79].

## Supporting Information

S1 File Variants found in all genes sets within the ASD cohort. (XLSX)

S2 File Variants found in all genes sets within the EVS cohort. (XLSX)

S3 File Odds ratio of neoplasm by gender and age computed from logistic regression with 2-factor interaction of autistic disorder with age and with gender (Figure A). Graphical depiction of the rates of diabetes mellitus in patients at UIHC with the diagnosis of autistic disorder vs. a control population (Figure B). Graphical depiction of the rates of essential hypertension in patients at UIHC with the diagnosis of autistic disorder vs. a control population (Figure C). Neoplasms found within patients with autism and within controls (Table A). Odds ratio estimates and wald confidence intervals for the effect of autistic disorder on cancer rate stratified by age and gender (Table B). Odds ratio estimates and wald confidence intervals for the additive effect of autism with age and sex on the log-odds of neoplasm (Table C). Tabular data for cancer rate in autism and other diagnoses (Table D).
(PPTX)

S1 Table Gene lists used in enrichment analysis. (XLSX)

S2 Table Identifiers of individuals within the Broad-generated VCF file (c1_NIMH_Autism_HGSC_Broad_broad.vcf) with a diagnosis of autism, excluding non-affected parental samples. (XLSX)
