# Supplementary material for: Autism Linked to Increased Oncogene Mutations but Decreased Cancer Rate
Source: PLoS One. 2016 Mar 2;11(3):e0149041. doi: 10.1371/journal.pone.0149041 (PMC4774916; doi:10.1371/journal.pone.0149041)
Supplement: S3 File — Odds ratio of neoplasm by gender and age computed from logistic regression with 2-factor interaction of autistic disorder with age and with gender (Figure A). Graphical depiction of the rates of diabetes mellitus in patients at UIHC with the diagnosis of autistic disorder vs. a control population (Figure B). Graphical depiction of the rates of essential hypertension in patients at UIHC with the diagnosis of autistic disorder vs. a control population (Figure C). Neoplasms found within patients with autism and within controls (Table A). Odds ratio estimates and wald confidence intervals for the effect of autistic disorder on cancer rate stratified by age and gender (Table B). Odds ratio estimates and wald confidence intervals for the additive effect of autism with age and sex on the log-odds of neoplasm (Table C). Tabular data for cancer rate in autism and other diagnoses (Table D). (PPTX) [file pone.0149041.s003.pptx]

## Slide 1
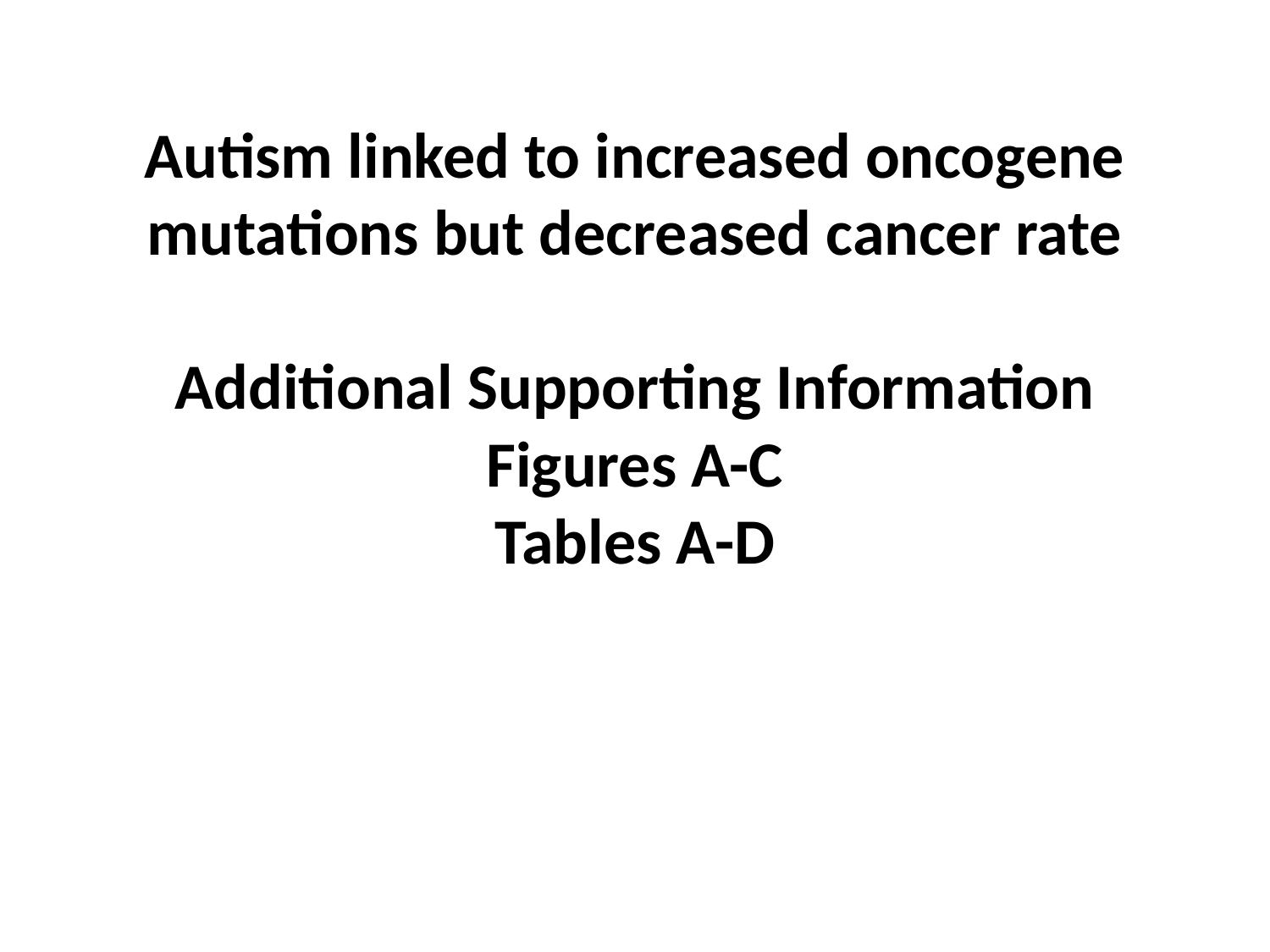

# Autism linked to increased oncogene mutations but decreased cancer rate Additional Supporting Information Figures A-C Tables A-D

## Slide 2
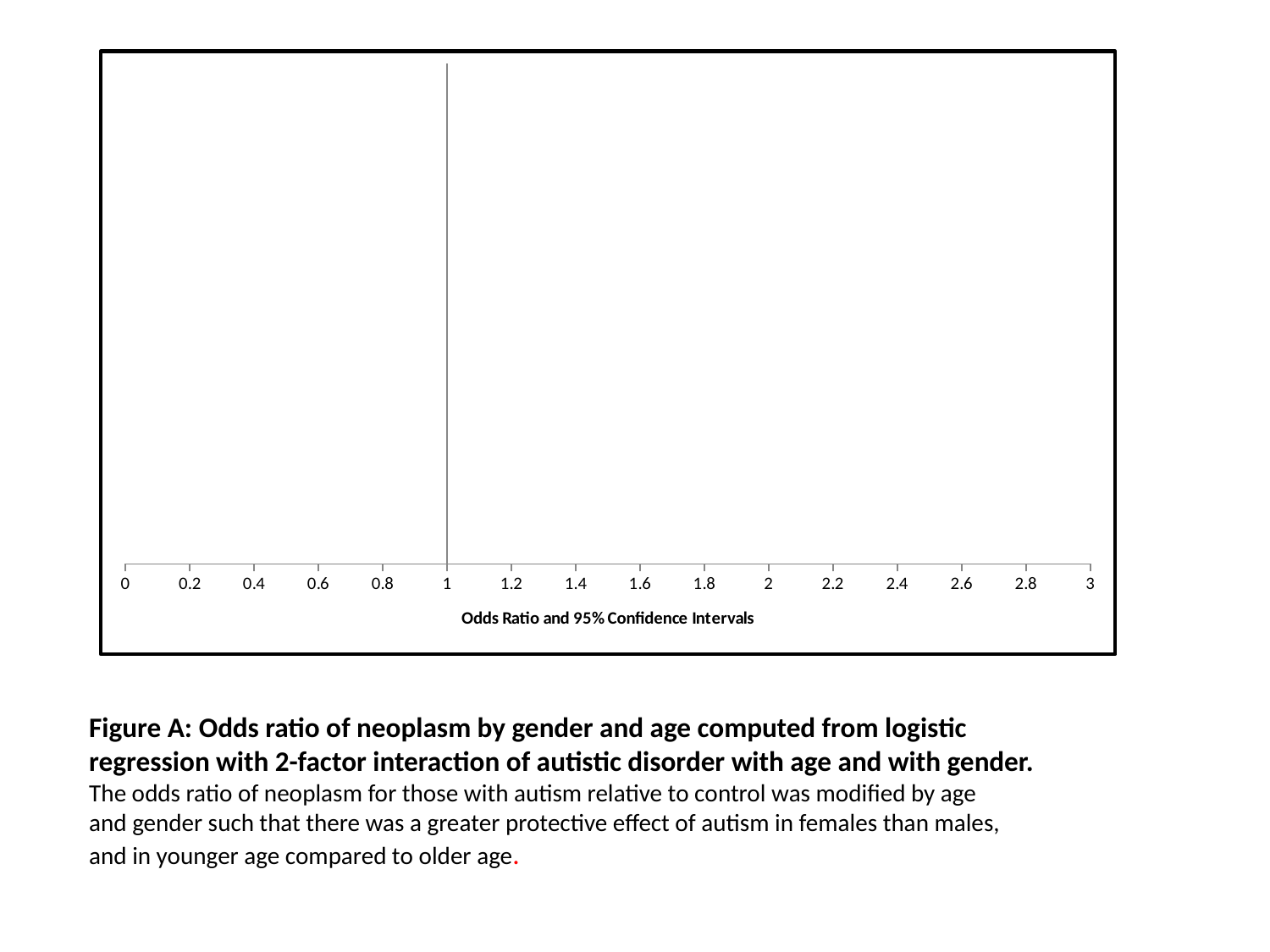

### Chart
| Category | | |
|---|---|---|Figure A: Odds ratio of neoplasm by gender and age computed from logistic
regression with 2-factor interaction of autistic disorder with age and with gender.
The odds ratio of neoplasm for those with autism relative to control was modified by age
and gender such that there was a greater protective effect of autism in females than males,
and in younger age compared to older age.

## Slide 3
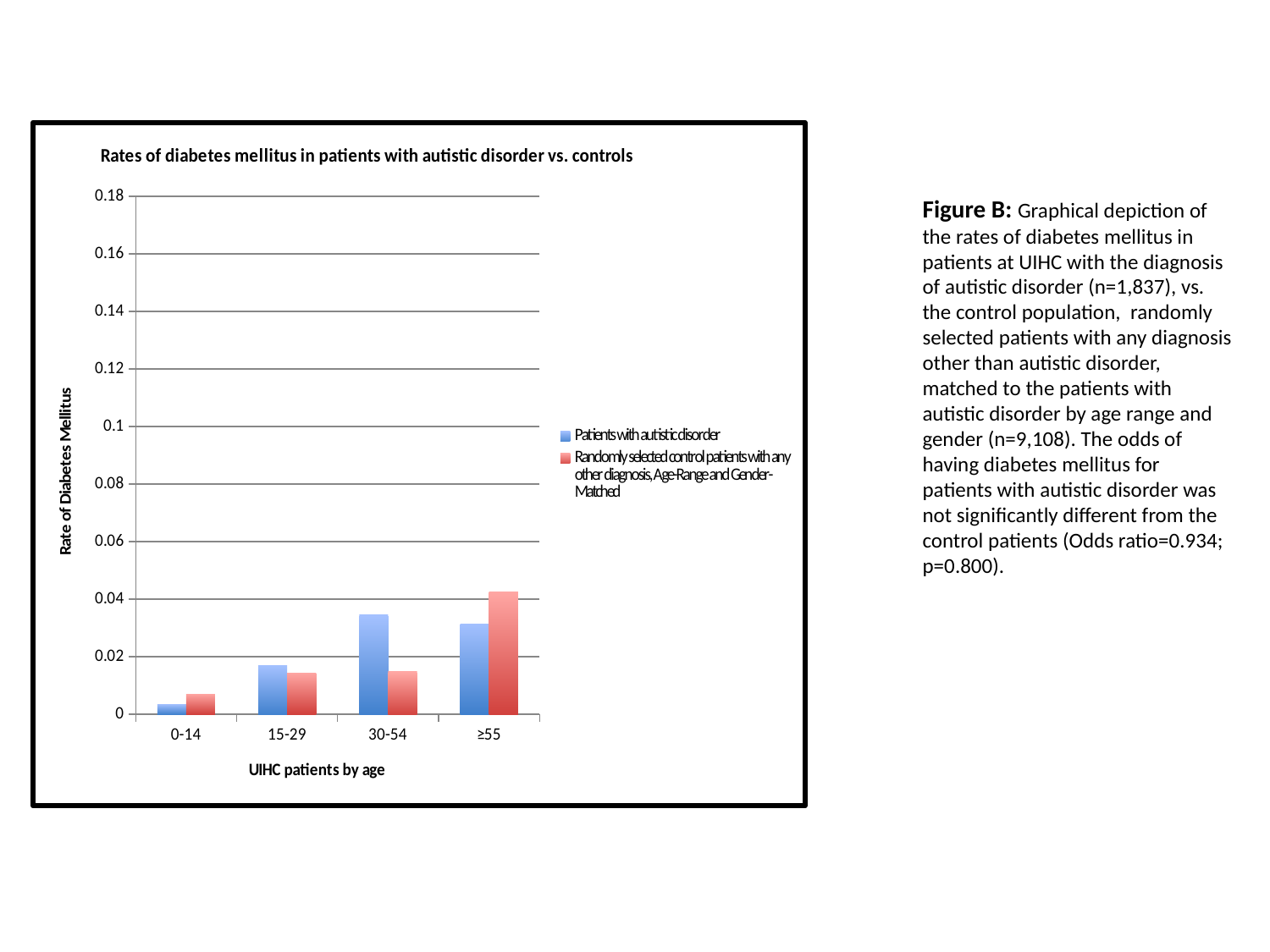

### Chart: Rates of diabetes mellitus in patients with autistic disorder vs. controls
| Category | Patients with autistic disorder | Randomly selected control patients with any other diagnosis, Age-Range and Gender- Matched |
|---|---|---|
| 0-14 | 0.00327868852459016 | 0.00699300699300699 |
| 15-29 | 0.0170575692963753 | 0.0142163661581137 |
| 30-54 | 0.0344827586206896 | 0.0149501661129568 |
| ≥55 | 0.03125 | 0.0425531914893617 |Figure B: Graphical depiction of the rates of diabetes mellitus in patients at UIHC with the diagnosis of autistic disorder (n=1,837), vs. the control population, randomly selected patients with any diagnosis other than autistic disorder, matched to the patients with autistic disorder by age range and gender (n=9,108). The odds of having diabetes mellitus for patients with autistic disorder was not significantly different from the control patients (Odds ratio=0.934; p=0.800).

## Slide 4
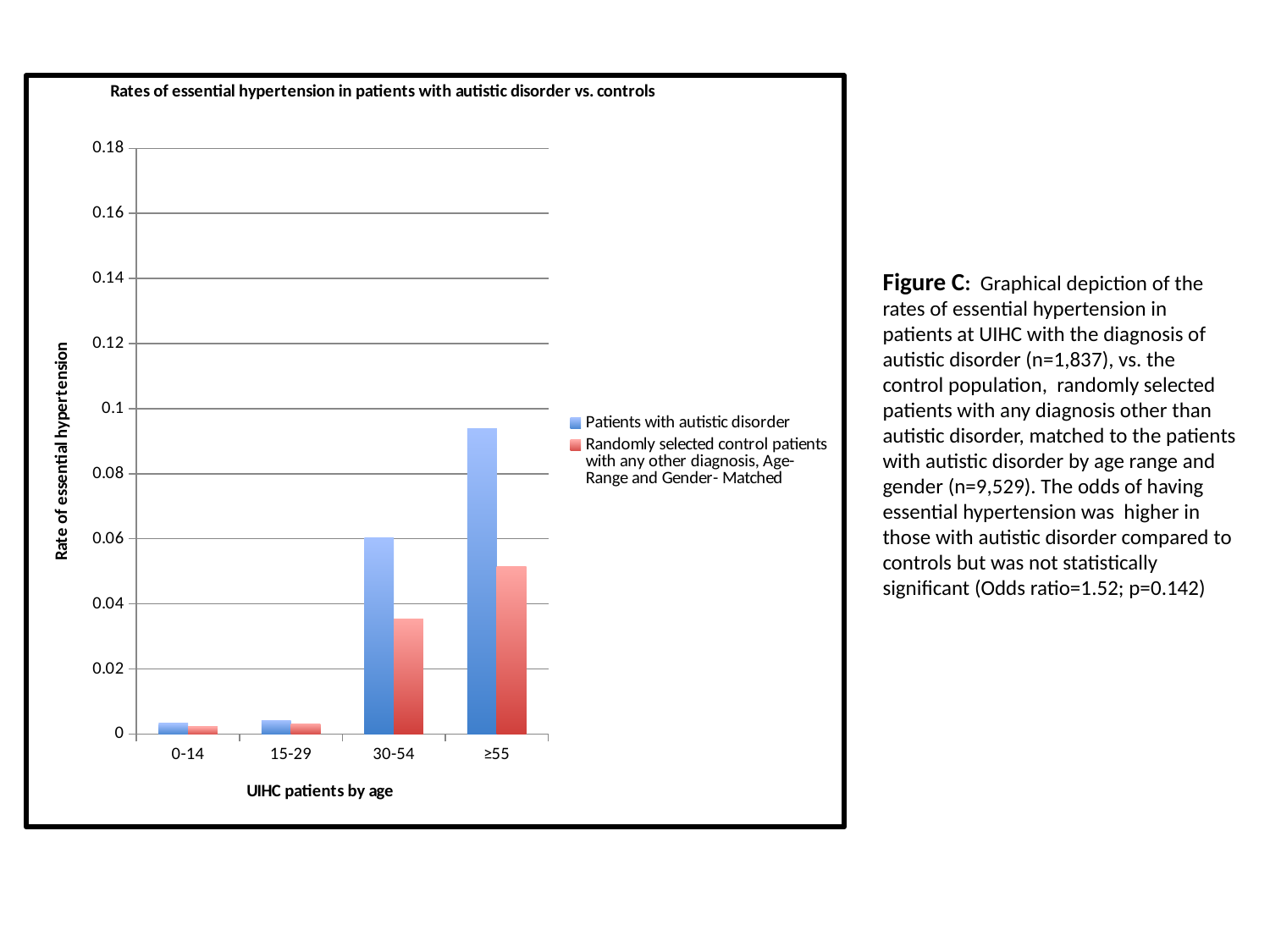

### Chart: Rates of essential hypertension in patients with autistic disorder vs. controls
| Category | Patients with autistic disorder | Randomly selected control patients with any other diagnosis, Age-Range and Gender- Matched |
|---|---|---|
| 0-14 | 0.00327868852459016 | 0.00239852398523985 |
| 15-29 | 0.00426439232409382 | 0.00311850311850312 |
| 30-54 | 0.0603448275862069 | 0.0353053435114504 |
| ≥55 | 0.09375 | 0.0514285714285714 |Figure C: Graphical depiction of the rates of essential hypertension in patients at UIHC with the diagnosis of autistic disorder (n=1,837), vs. the control population, randomly selected patients with any diagnosis other than autistic disorder, matched to the patients with autistic disorder by age range and gender (n=9,529). The odds of having essential hypertension was higher in those with autistic disorder compared to controls but was not statistically significant (Odds ratio=1.52; p=0.142)

## Slide 5
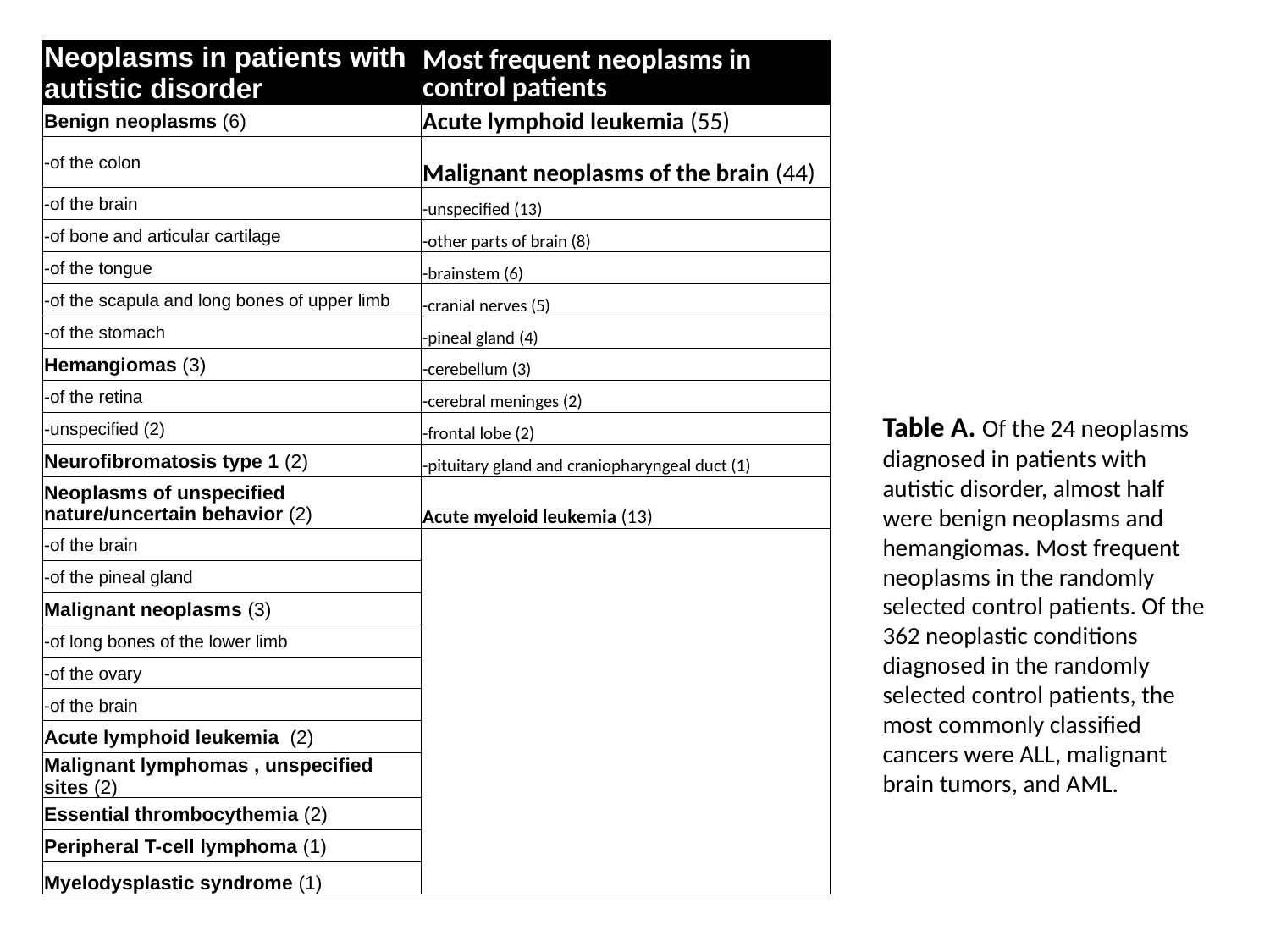

| Neoplasms in patients with autistic disorder | Most frequent neoplasms in control patients |
| --- | --- |
| Benign neoplasms (6) | Acute lymphoid leukemia (55) |
| -of the colon | Malignant neoplasms of the brain (44) |
| -of the brain | -unspecified (13) |
| -of bone and articular cartilage | -other parts of brain (8) |
| -of the tongue | -brainstem (6) |
| -of the scapula and long bones of upper limb | -cranial nerves (5) |
| -of the stomach | -pineal gland (4) |
| Hemangiomas (3) | -cerebellum (3) |
| -of the retina | -cerebral meninges (2) |
| -unspecified (2) | -frontal lobe (2) |
| Neurofibromatosis type 1 (2) | -pituitary gland and craniopharyngeal duct (1) |
| Neoplasms of unspecified nature/uncertain behavior (2) | Acute myeloid leukemia (13) |
| -of the brain | |
| -of the pineal gland | |
| Malignant neoplasms (3) | |
| -of long bones of the lower limb | |
| -of the ovary | |
| -of the brain | |
| Acute lymphoid leukemia (2) | |
| Malignant lymphomas , unspecified sites (2) | |
| Essential thrombocythemia (2) | |
| Peripheral T-cell lymphoma (1) | |
| Myelodysplastic syndrome (1) | |
Table A. Of the 24 neoplasms diagnosed in patients with autistic disorder, almost half were benign neoplasms and hemangiomas. Most frequent neoplasms in the randomly selected control patients. Of the 362 neoplastic conditions diagnosed in the randomly selected control patients, the most commonly classified cancers were ALL, malignant brain tumors, and AML.

## Slide 6
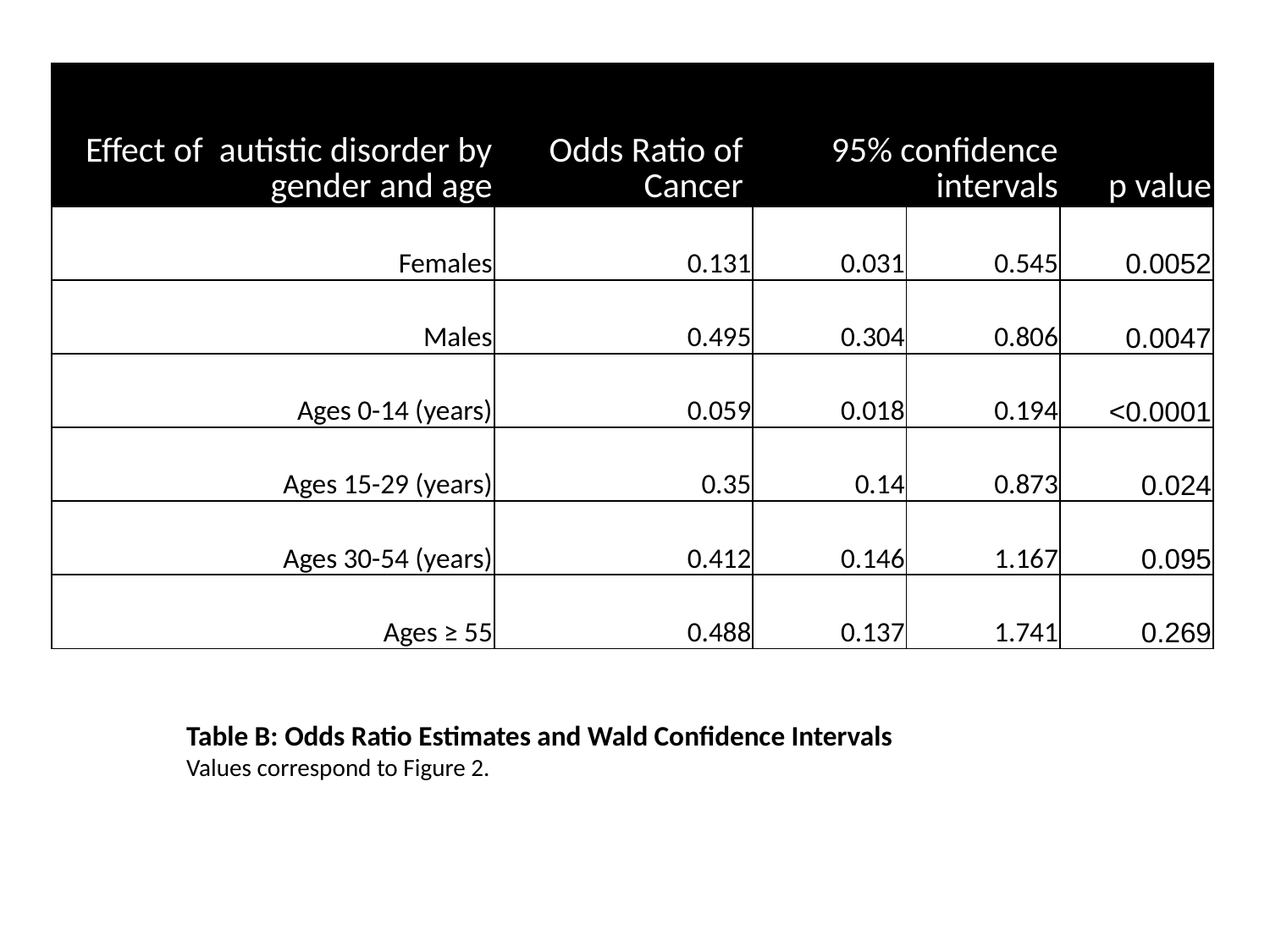

| Effect of autistic disorder by gender and age | Odds Ratio of Cancer | 95% confidence intervals | | p value |
| --- | --- | --- | --- | --- |
| Females | 0.131 | 0.031 | 0.545 | 0.0052 |
| Males | 0.495 | 0.304 | 0.806 | 0.0047 |
| Ages 0-14 (years) | 0.059 | 0.018 | 0.194 | <0.0001 |
| Ages 15-29 (years) | 0.35 | 0.14 | 0.873 | 0.024 |
| Ages 30-54 (years) | 0.412 | 0.146 | 1.167 | 0.095 |
| Ages ≥ 55 | 0.488 | 0.137 | 1.741 | 0.269 |
Table B: Odds Ratio Estimates and Wald Confidence Intervals
Values correspond to Figure 2.

## Slide 7
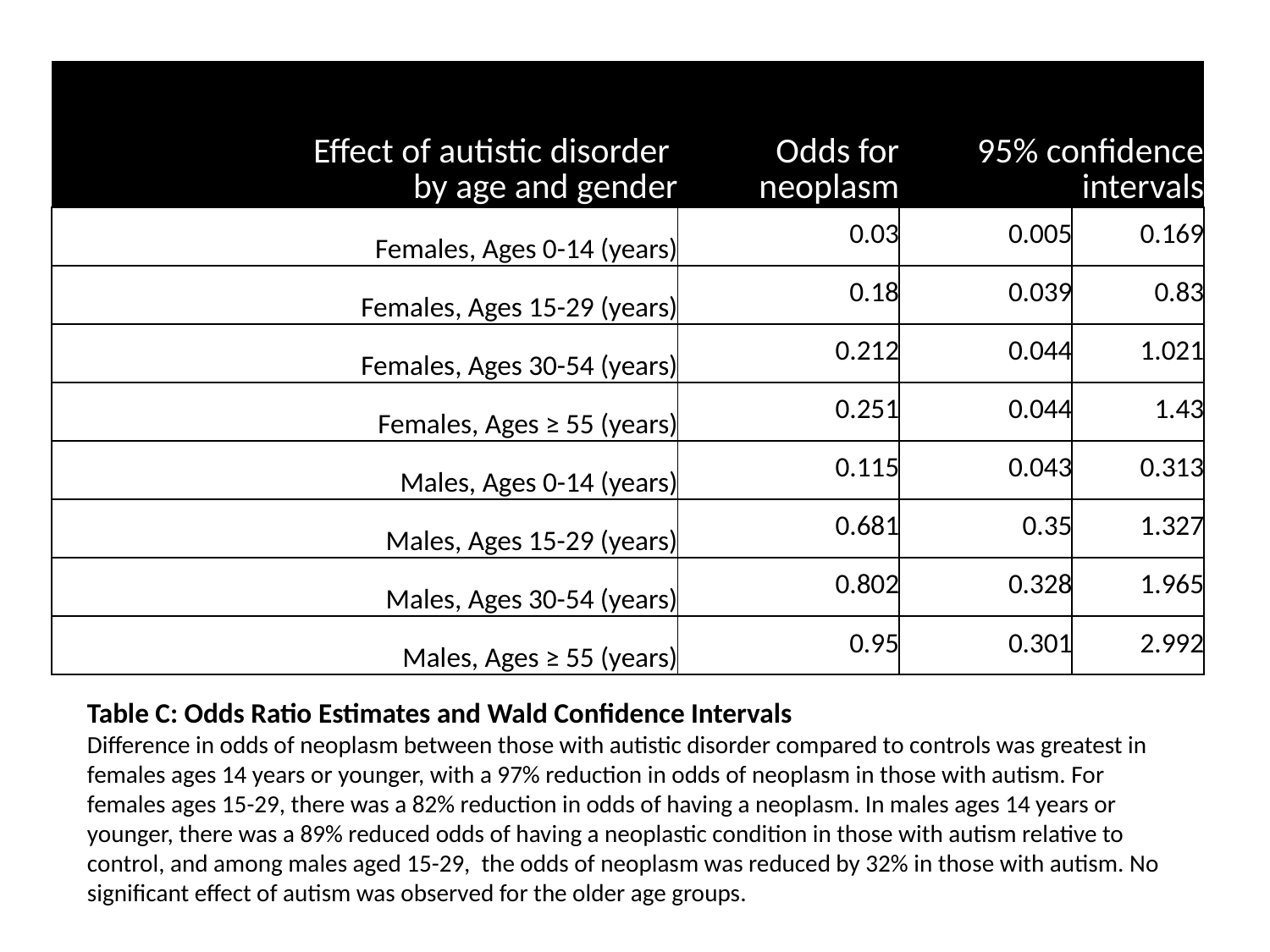

| Effect of autistic disorder by age and gender | Odds for neoplasm | 95% confidence intervals | |
| --- | --- | --- | --- |
| Females, Ages 0-14 (years) | 0.03 | 0.005 | 0.169 |
| Females, Ages 15-29 (years) | 0.18 | 0.039 | 0.83 |
| Females, Ages 30-54 (years) | 0.212 | 0.044 | 1.021 |
| Females, Ages ≥ 55 (years) | 0.251 | 0.044 | 1.43 |
| Males, Ages 0-14 (years) | 0.115 | 0.043 | 0.313 |
| Males, Ages 15-29 (years) | 0.681 | 0.35 | 1.327 |
| Males, Ages 30-54 (years) | 0.802 | 0.328 | 1.965 |
| Males, Ages ≥ 55 (years) | 0.95 | 0.301 | 2.992 |
Table C: Odds Ratio Estimates and Wald Confidence Intervals
Difference in odds of neoplasm between those with autistic disorder compared to controls was greatest in females ages 14 years or younger, with a 97% reduction in odds of neoplasm in those with autism. For females ages 15-29, there was a 82% reduction in odds of having a neoplasm. In males ages 14 years or younger, there was a 89% reduced odds of having a neoplastic condition in those with autism relative to control, and among males aged 15-29, the odds of neoplasm was reduced by 32% in those with autism. No significant effect of autism was observed for the older age groups.

## Slide 8
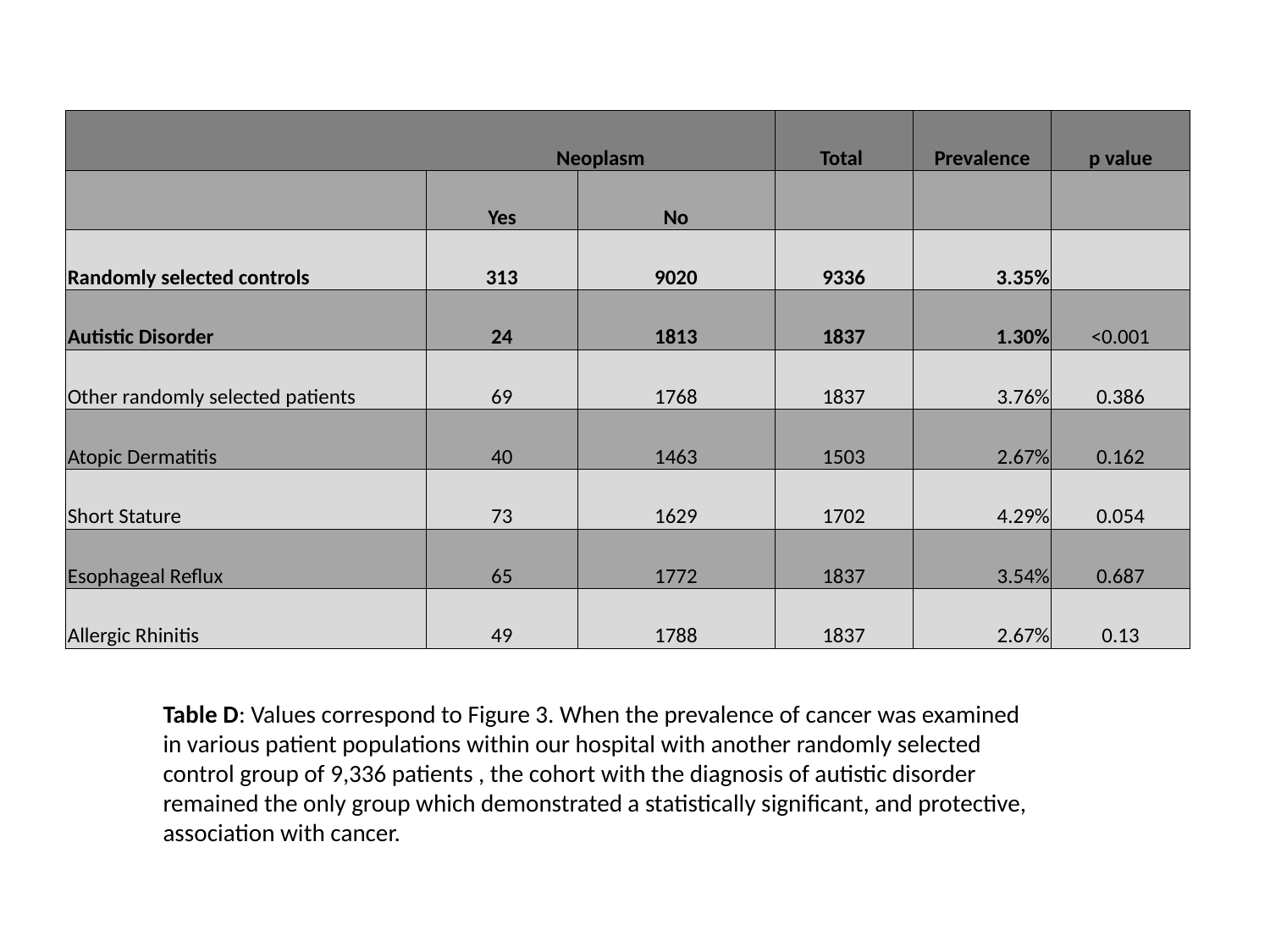

| | Neoplasm | | Total | Prevalence | p value |
| --- | --- | --- | --- | --- | --- |
| | Yes | No | | | |
| Randomly selected controls | 313 | 9020 | 9336 | 3.35% | |
| Autistic Disorder | 24 | 1813 | 1837 | 1.30% | <0.001 |
| Other randomly selected patients | 69 | 1768 | 1837 | 3.76% | 0.386 |
| Atopic Dermatitis | 40 | 1463 | 1503 | 2.67% | 0.162 |
| Short Stature | 73 | 1629 | 1702 | 4.29% | 0.054 |
| Esophageal Reflux | 65 | 1772 | 1837 | 3.54% | 0.687 |
| Allergic Rhinitis | 49 | 1788 | 1837 | 2.67% | 0.13 |
Table D: Values correspond to Figure 3. When the prevalence of cancer was examined in various patient populations within our hospital with another randomly selected control group of 9,336 patients , the cohort with the diagnosis of autistic disorder remained the only group which demonstrated a statistically significant, and protective, association with cancer.
